# Hurdles to Cardioprotection in the Critically Ill

**DOI:** 10.3390/ijms20153823

**Published:** 2019-08-05

**Authors:** Louise E See Hoe, Nicole Bartnikowski, Matthew A Wells, Jacky Y Suen, John F Fraser

**Affiliations:** 1 Critical Care Research Group, The Prince Charles Hospital, Chermside 4032, Australia; 2 Faculty of Medicine, University of Queensland, Chermside 4032, Australia; 3 Science and Engineering Faculty, Queensland University of Technology, Chermside 4032, Australia; 4 School of Medical Science, Griffith University, Southport 4222, Australia

**Keywords:** heart failure, heart transplantation, mechanical circulatory support, cardioprotection

## Abstract

Cardiovascular disease is the largest contributor to worldwide mortality, and the deleterious impact of heart failure (HF) is projected to grow exponentially in the future. As heart transplantation (HTx) is the only effective treatment for end-stage HF, development of mechanical circulatory support (MCS) technology has unveiled additional therapeutic options for refractory cardiac disease. Unfortunately, despite both MCS and HTx being quintessential treatments for significant cardiac impairment, associated morbidity and mortality remain high. MCS technology continues to evolve, but is associated with numerous disturbances to cardiac function (e.g., oxidative damage, arrhythmias). Following MCS intervention, HTx is frequently the destination option for survival of critically ill cardiac patients. While effective, donor hearts are scarce, thus limiting HTx to few qualifying patients, and HTx remains correlated with substantial post-HTx complications. While MCS and HTx are vital to survival of critically ill cardiac patients, cardioprotective strategies to improve outcomes from these treatments are highly desirable. Accordingly, this review summarizes the current status of MCS and HTx in the clinic, and the associated cardiac complications inherent to these treatments. Furthermore, we detail current research being undertaken to improve cardiac outcomes following MCS/HTx, and important considerations for reducing the significant morbidity and mortality associated with these necessary treatment strategies.

## 1. Introduction

Cardiovascular disease (CVD) accounts for approximately one third of total global deaths, is the primary cause of death in people aged > 15 years old, and is heavily impacted by global population growth and ageing [1]. Heart failure (HF) affects over 26 million people globally [2]. In the US, it is projected that by 2030, HF prevalence will have almost doubled since 2013, and is estimated to reach ~$70 billion in healthcare costs [3,4]. The 20th century gave rise to extensive developments in diagnostics, mechanical circulatory support (MCS) technology, pharmacotherapeutics, and strategies aimed at delaying or reversing cardiac injury in various planned and unplanned circumstances [5]. Survival from HF continues to improve in response to effective treatments for HF risk factors, pharmacological approaches (e.g., β-blockers, angiotensin-converting enzyme inhibitors, statins), and percutaneous coronary interventions. However, this trend is not equal across all demographics [6].

Hospitalization following HF diagnosis is common, with one study reporting that 43% of patients were hospitalized four times following diagnosis [7]. Indeed, HF is the primary cause for hospital admission in those aged >65 years (US) [8]. Following hospitalization, the causes of death from HF or rehospitalisation vary significantly. For end-stage or refractory HF, there is no cure, and heart transplantation (HTx) is the final option available to clinicians. Depending on the country and respective regulations, critically ill cardiac patients may be amenable to differing forms of MCS, allowing time for treating physicians to make a decision, recovery of the heart prior to further extensive intervention, or as a destination therapy. The common forms of MCS include ventricular assist devices (VADs) and extracorporeal membrane oxygenation (ECMO).

The chronically ill cardiac patient has likely experienced accidental or sudden (e.g., Non-surgical ischemia or acute myocardial infarction) or surgical ischemia (e.g., cardiopulmonary bypass) as a result of cardiac complications, thus exposing them to ischemia-reperfusion injury (IRI). The pathophysiological mechanisms of IRI have been extensively detailed and reviewed elsewhere [9,10,11]. Due to the persistent and increasing burden of CVD, cardioprotective strategies aimed at limiting infarct size and IRI perturbations have been intensely investigated for almost 50 years. The NHLBI Working Group on the Translation of Therapies for Protecting the Heart from Ischemia stated in 2004 that ‘three decades after its birth, the protection of the myocardium in the setting of AMI remains an unfulfilled promise’ [12]. We are approaching 50 years since cardioprotective research began, and there is still no change [13,14].

Several cardioprotective phenomena have been assessed extensively in various settings in both animals and humans. Brief intermittent episodes of myocardial ischemia-reperfusion (IR) either prior to or following a period of extended ischemia to limit infarct size are termed ischemic pre- and postconditioning, respectively. Initiating the repetitive cycles of brief IR in a remote organ or tissue to positively influence myocardial recovery from a sustained period of ischemia describes remote ischemic conditioning, which can be applied before, after or during the index ischemia. Ischemic pre-, post-, per-conditioning, and remote conditioning strategies initiate innate protective signaling mechanisms to improve post-ischemic myocardial recovery. Mechanistically, this generally involves activation of G-protein-coupled receptors (GPCRs) to initiate a cascade of pro-survival signaling pathways (typically Reperfusion Injury Salvage Kinase, RISK; or Survivor Activator Factor Enhancement, SAFE) that converge upon mitochondrial targets to preserve mitochondrial function, and promote the transcription of protective genes, thus reducing cellular injury [15,16,17]. Similar responses can be derived pharmacologically using ligands that activate these pathways or components thereof (e.g., adenosine, opioids) [9,16,17]. Numerous cardioprotective strategies have shown significant promise in small animal and preclinical studies, yet studies in human trials have all yielded generally poor outcomes. This failure in effective clinical translation has been attributed to shortfalls in design and conduct of both animal and clinical studies, with animal studies failing to account for relevant target cohort characteristics (e.g., comorbidities, medications, age, cardiovascular risk factors), and clinical trials facing issues with dosing and timing of the desired cardioprotective therapeutic, and appropriate patient selection [13,14,16]. 

As the use of MCS technology continues to increase exponentially, in a health service that is crumbling under the increasing ageing population, it is naïve to believe this will not impact the incidence of refractory cardiac patients requiring treatment. Our research group has developed several preclinical models investigating MCS (ventricular assist devices, VADs) [18], extra-corporeal membrane oxygenation (ECMO) [19], and HTx [20]. These are important life-saving interventions used for patients with severe cardiac damage. While cardioprotection has traditionally referred to limiting the effects of IRI, in the context of this review, cardioprotection also refers to different strategies aiming to reduce the detrimental effects of cardiac surgical interventions (that may include IRI). Looking forward, this review will detail the important considerations for critically ill cardiac patients in the context of MCS and HTx for effective cardioprotection to be beneficial and effective in this cohort, and if research has unveiled any potentially effective therapeutics.

## 2. Critically Ill Cardiac Patients 

End-stage HF is a progressive and complex clinical syndrome that results from impaired cardiac function to sufficiently meet physiological demands, leading to vascular congestion and tissue hypoperfusion [21,22]. Heart failure can develop following various cardiovascular disturbances such as AMI, myocarditis, metabolic syndrome, drug/alcohol abuse, arrhythmia, hypertension, cardiomyopathy [21,22,23]. Cardiac dysfunction is a hallmark of HF and the heart is exposed to extensive adverse remodeling that further exacerbates the myocardial impairment. However, the extensive cardiac dysfunction broadens to impair the systemic vasculature, with significant activation of neurohormonal and/or inflammation pathways, culminating in severe end-organ dysfunction (e.g., anemia, renal failure, cachexia, respiratory complications) [22,24]. Several recent studies have highlighted the profound molecular changes upon the vasculature following AMI, that could be important potentiating factors for the development of HF. Using tissue microarray, Kong and colleagues demonstrated that collagen III is reduced in the aortic wall extracellular matrix of AMI patients, and suggested this reduction may enhance plaque vulnerability, and thus the propensity for vessel occlusion and AMI [25]. Modification of membrane lipids is an important factor in the development of cellular injury, tolerance to ischemia and dependence of cardioprotective/cytoprotective signaling [26,27]. Indeed, unique lipid profiling of ceramides in plasma (but not aortic tissue) following AMI, have recently been demonstrated to predict long-term incidence of detrimental cardiac and cerebrovascular events [28]. The authors suggested that this risk signature profile is of myocardial origin following ischemia, not from the vasculature [28]. AMI also induces specific transcriptome patterns in vascular smooth muscle cells (VSMCs) from the aortic wall. Gene expression analysis of VSMCs identified upregulation of hypoxia signaling in the cardiovascular system [29] and cadherin superfamily members [30]. These molecular myocardial and systemic alterations associated with AMI may be important considerations for the development and subsequent management of HF patients. 

## 3. Mechanical Circulatory Support (MCS)

The gold standard treatment of end-stage HF, heart transplantation (HTx), is limited by a lack of available donor organs. Consequently, MCS has become an important treatment strategy for severe acute and chronic HF [31,32]. The development of MCS systems have paralleled the expansion of HTx [32], and has continued to expand due to improved devices and clinical acceptance. 

**Figure 1:**
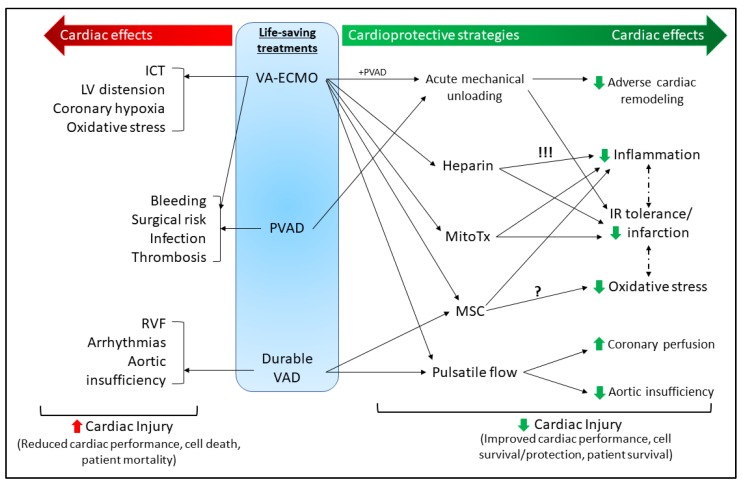
Summary of the current mechanical and molecular cardioprotective strategies associated with mechanical circulatory support MCS (discussed in Section 3). In the absence of effective conventional therapies for severe acute and chronic HF, the use of MCS (VA-ECMO, PVAD, durable VAD) can be a life-saving treatment for patients, but due to technical limitations and surgical risks, remains associated with a high risk of mortality (left side of figure in red). These MCS strategies can be used to provide a mechanical cardioprotective benefit (e.g., pulsatile flow, acute mechanical unloading); however, molecular cardioprotective strategies (e.g., MitoTx, MSC) are being investigated for use alongside these treatments to increase their cardiac benefit, reduce adverse side effects, and improve patient survival (right side of figure in green). Heparin is typically cardioprotective independently of its important anticoagulation role; however, in ECMO settings is associated with hemodynamic complications and limited patient survival (this risk is denoted by !!!). Additionally, while MSCs have demonstrated roles in limiting oxidative stress in various settings [34], this is yet to be delineated in settings of ECMO. VA-ECMO—venoarterial extracorporeal membrane oxygenation; PVAD—percutaneous ventricular assist device (VAD); ICT—intracardiac thrombosis; LV—left ventricular; RVF—right ventricle failure; MitoTx—mitochondrial transplant; MSC—mesenchymal stem cells; IR—ischemia-reperfusion.

MCS provides an attractive treatment option when conventional medical therapies fail. However, widespread use is still inhibited due to technological limitations, inherent complications with MCS, and suboptimal clinical application [33]. The MCS systems discussed herein are focused on those that can aid/provide potential treatment strategies but can implement cardioprotective treatments, or have shown some clinical promise for long-term cardiac recovery, including extracorporeal membrane oxygenation (ECMO) and ventricular assist devices (VADs) (summarized in Figure 1).

### 3.1. Short-Term MCS

Short-term MCS has evolved significantly as an effective method of acute support for patients with cardiogenic shock, which still has an extremely high mortality rate at 50% [35,36,37]. Short-term MCS devices are used as either a bridge to myocardial recovery, long-term MCS or HTx, and can also be used to aid high-risk coronary interventions [38,39,40]. Recent technological advances have dramatically improved the efficacy of short-term MCS devices, leading to use in an earlier stage of HF, in a broader number of patients. Options for short-term MCS that could further benefit patients by providing cardioprotective treatment include ECMO and percutaneous ventricular assist devices (PVADs), such as the TandemHeart^®^ and Impella^®^ systems [41]. 

#### 3.1.1. Extracorporeal Membrane Oxygenation (ECMO)

ECMO is a modified form of the cardiopulmonary bypass device used to support patients in critical conditions with life-threatening respiratory failure or cardiogenic shock. Through the use of a continuous flow, centrifugal pump and a membrane oxygenator, ECMO drains deoxygenated, venous blood from the patient through a large cannula, externally reoxygenates blood in the oxygenator, and returns oxygenated blood via another cannula into the body. The choice of ECMO cannulation (type and size of cannula) significantly impacts cardiopulmonary function whilst on ECMO, and is an important consideration to achieve maximal hemodynamic support whilst limiting ECMO-associated complications (e.g., infection, bleeding, stroke, thrombosis). The resultant ECMO configuration (specific venous and arterial placement of cannulas throughout the body) provides temporary hemodynamic support, but must be considered dynamic, and ECMO cannulation strategies and configurations must evolve with the changing patient requirements to achieve optimal outcomes [42]. The technology has existed for >40 years and its use has seen significant growth over the last decade (2008: 2803 cases vs 2018: 10,423 cases) [43]. In particular, veno-arterial (V-A) ECMO has been used successfully for various cardiac diseases, including cardiogenic shock, myocarditis, acute coronary syndrome, cardiomyopathy, cardiac arrest refractory to usual resuscitative techniques, bridge to long-term MCS or HTx, primary graft failure, and secondary HTx rejection. Unfortunately, ECMO is associated with a high mortality (40%–60%) [44,45]. Most V-A ECMO research has been physiological in nature, examining the effects and potential benefits on patients, particularly those with cardiogenic shock. In contrast, few studies have examined the molecular and mechanistic basis of ECMO-associated adverse effects on patients. 

#### 3.1.2. Percutaneous VADs

PVADs have been very effective in treating patients with cardiogenic shock as a bridge to recovery, by improving the hemodynamic stability of the patient and increasing survival by reducing the risk of multisystem organ failure [46]. There has been substantial growth in the use of PVADs clinically, with a 30× increase in PVAD implants between 2007 and 2012 [44,45], coupled with a decrease in intra-aortic balloon pump (IABP) usage over the same time period. PVADs are able to provide robust hemodynamic support with a cardiac output up to 4–5 L·min^−1^, compared to IABPs which can only provide 0.5 L·min^−1^ of support [36,44]. Clinically available PVADs include the Impella^®^ (Abiomed, Inc., Danvers, MA, USA) or the TandemHeart^®^ (CardiacAssist, Inc., Pittsburgh, Pennsylvania).

The TandemHeart system consists of a 21F venous transeptal inflow cannula, implanted through the femoral vein and right atrium, which withdraws oxygenated blood from the left atrium (LA) [47]. This is then pumped into the systemic circulation via a 17F femoral artery catheter using a continuous flow centrifugal blood pump and an arterial perfusion catheter [41], with a flow rate of 4 L·min^−1^. There have been two randomized clinical studies comparing the TandemHeart to IABP in patients with cardiogenic shock, secondary to acute myocardial infarction [48,49]. These studies demonstrated superior LV unloading and improved end-organ perfusion compared to the IABP. However, neither study reported a difference in 30-day mortality, with the use of larger insertion cannulas for the TandemHeart resulting in more bleeding and limb ischemia [49]. 

The Impella devices are catheter mounted continuous flow axial blood pumps which are placed across the aortic valve [46], with the inflow of the cannula within the left ventricle (LV) and the outflow pumping into the aorta. There are several versions of the Impella pump that differ in size, implantation method and output. The Impella 2.5 (12F) is the most commonly used pump and along with the Impella CP (14F), this device is implanted percutaneously, with a 2.5 L·min^−1^ and 3 L·min^−1^ flow, respectively [41]. Due to the ease of implantation, the Impella 2.5 and CP are frequently used to support the heart during interventions such as revascularization and ablation [50]. The larger versions of this device, the Impella 5.0 (21F) and LD (21F) both require surgical cutdown, but have a higher flow rate of 5 L min^−1^ [41]. Similarly to the TandemHeart clinical trials, Seyfarth et al. [51] reported in the ISAR-SHOCK trial comparing Impella 2.5 to IABP for patients with cardiogenic shock, that the Impella 2.5 provided superior hemodynamic support, however there was no difference in 30-day mortality. Similarly, retrospective analysis by Schrage et al. identified no improvement in 30-day mortality following Impella use in patients with AMI complicated by cardiogenic shock [52]. Both the TandemHeart and the Impella have been shown clinically to unload the LV while improving systemic blood flow, despite the different hemodynamic modes of these pumps [49,51,53]. Both of these PVADs offer strategic therapeutic options for mechanically unloading the heart, a recently evolving clinical cardioprotective strategy, while causing minimal complications compared to other MCS systems. 

#### 3.1.3. Durable VADs

Durable VADs are rotary blood pumps that have been developed to support the failing heart and restore blood flow to the body, by removing blood from the LV and returning it into the circulatory system via the aorta [54,55]. The size, durability and reliability of VADs have progressed substantially in the past decade, emerging as the main device for providing short to long-term support of a failing LV for patients awaiting HTx. However as these devices have evolved as a standard treatment for end-stage HF patients, VADs are now being used as a destination therapy for patients ineligible for HTx [56,57]. As a result, there has been a substantial increase in the number of VADs implanted in the last five years, with approximately 46% of all implants as a destination therapy. According to the USA’s Interagency Registry for Mechanical Assisted Circulatory Support (INTERMACS) [58], there was an >25x increase in device implantations from 95 in 2006, to over 2500 in 2016. 

VAD implantation has also been targeted toward bridge to myocardial recovery, with a limited number of patients thus far able to recover sufficient cardiac function to be weaned from the device [59]. By their design, VADs remove excess mechanical load on the heart, thereby allowing the heart to potentially recover some myocardial structure and function. A disconnect persists between biological recovery and improved clinical outcomes that require further investigation. According to the INTERMACS database, of patients who received VADs from 2006–2013, only 0.9% were bridged to recovery [60]. However, durable VADs may prove to be a more common therapeutic approach in the future with advancing technology, and with a large subset of patients reliant on these devices as a destination therapy [61]. 

The HeartMate II (Thoratec Corporation, Pleasanton, CA) is the most widely implanted VAD, with over 20,000 devices implanted worldwide [60]. It is an axial continuous flow pump that gained FDA approval for bridge to transplant (BTT) in 2008 and as a destination therapy in 2010. The next generation device, the HeartMate 3, is a centrifugal continuous flow pump that includes a small artificial pulse. The MOMENTUM 3 trial demonstrated superiority in this pump compared with the HeartMate II device, with 77.9% of the HeartMate 3 patients surviving to 2 years post-implant without a disabling stroke, or need to replace the pump compared to only 56.4% of the HeartMate II patients [62]. The HeartMate 3 gained FDA approval as a BTT device in 2017 and as a destination therapy in 2018. 

### 3.2. MCS-Associated Complications

While the development of MCS systems have revolutionized the care of HF and critically ill patients, they are prone to complications that limits the use of these devices clinically. This will inevitably impede their clinical application as a cardioprotective strategy until these complications can be mitigated.

#### 3.2.1. ECMO Complications

ECMO has been associated with several systemic complications, including bleeding and thrombosis, infection, acute kidney injury, and neurologic complications and stroke [63,64,65]. There are also cardio-specific complications that are strongly associated with V-A ECMO, such as intracardiac thrombosis, coronary hypoxia, LV distention and oxidative stress, which will be the focus of the review herein. These complications along, with the high mortality rate associated with this treatment, are a significant barrier to increased use of ECMO and its implementation in the ICU as a cardioprotective strategy. 

Cardiac Thrombosis - Intracardiac thrombosis (ICT) is a well-recognized complication for V-A ECMO. Though rare, it can be life-threatening and several case reports have highlighted the significant mortality associated with ICT on V-A ECMO [66,67]. Williams and Bernstein summarized 12 reported cases of ICT, where the primary indication for V-A ECMO was cardiogenic shock, secondary to myocardial infarction [68]. Heparin was used and reported in 8 of these cases. Of the 12 patients with ICT, only two patients survived. Similarly, Weber et al. retrospectively analyzed their femoral V-A ECMO patients between 2007 and 2015, and identified 11 patients (3.91%) developed intra- or extra-cardiac thrombus [69]. In this study, approximately 50% of patients received V-A ECMO post-cardiotomy, while the other 50% were supported with V-A ECMO as extracorporeal cardiopulmonary resuscitation. All patients received heparin anticoagulation, with ACT (activated clotting time) and aPTT (activated thromboplastin time) monitoring. No patients survived in this cohort. Despite receiving anticoagulation (traditionally with intravenous unfractionated heparin), thrombosis continues to be a major issue for ECMO patients in general. Development of LV thrombosis is present in up to 40% of patients presenting with AMI and ventricular dysfunction, as decreased contractility of the ventricles greatly increased potential for intracavitary blood stasis. This is further exacerbated with the retrograde flow of V-A ECMO, which significantly increases afterload and impairs the ejection of blood from the LV, leading to closure of the aortic valve. Currently, minimal evidence exists to guide the best approach to treat V-A ECMO patients with ICT. Treatment modalities used for ICT management include surgical thrombectomy, local thrombolysis and use of VAD to ensure adequate forward flow of blood [68,70].

Left ventricular distention- During V-A ECMO, the retrograde flow causes increased LV afterload and pressure in the aorta. This can lead to LV distension as the impaired LV is unable to generate sufficient power to overcome the increased afterload in order to eject blood. This causes progressive LV dysfunction, wall stress, pulmonary edema, and impairment of myocardial oxygenation [71,72]. In order to address these complications, a number of strategies to facilitate LV unloading are suggested. These include pharmacological approaches, inotropic support, IABP implantation, surgical LV venting, and Impella implantation [73].

Coronary Hypoxia - Oxygen saturation on V-A ECMO is usually normal (>90%), as the upper body is provided with blood from LV that has been oxygenated by normally functioning lungs, while the lower body is supported by fully saturated blood from V-A ECMO [74]. However, in the event of patients with concomitant pulmonary dysfunction, blood ejected from the heart is no longer sufficiently oxygenated This deoxygenated blood preferentially supplies the coronary arteries, cerebral circulation and upper limbs, resulting in “dual circuits” and possible hypoxic injury (coronary and cerebral) [75,76]. This issue is well-supported by both preclinical studies and clinical cases, and most ECMO clinicians monitor for differential hypoxia [76,77]. Possible strategies to counteract this hypoxia include alternative cannulation strategy (V-AV ECMO) [76] and the recent development of pulsatile ECMO flow (to be discussed below). 

Oxidation Damage – Continuous exposure of blood to a foreign surface occurs with MCS, particularly with ECMO, and not only leads to an inflammatory response [78], but coupled with the on-going hyperoxia from the unnaturally high concentration of oxygen [79], causes an oxidant stress response and oxidative damage [80]. Patients supported by ECMO are susceptible to reactive nitrogen and oxygen species (RNOS). Lipid peroxidation, oxidative protein and DNA damage are all possible adverse consequences, and these could lead to altered membrane fluidity, permeability and subsequently physiological changes such as ion gradients, ultimately forming a positive feedback loop leading to more RNOS. Cardiomyocyte membrane integrity is integral to proper cardiac function, stress resistance and mechanisms of cardioprotection [26,27]. However, membrane integrity is also adversely impacted by age, metabolic disorders and IRI [26,27]. Thus, exaggerated oxidative damage upon an already at-risk heart (and cardiomyocyte membrane) could significantly exacerbate cardiac injury beyond repair. Oxidative damage is also a significant problem for MCS in general, and is a major contributor to complications such as postoperative atrial fibrillation, acute kidney injury and acute lung injury after cardiac surgery. Ultimately, oxidative stress is a key factor to ECMO patient mortality and morbidity. 

#### 3.2.2. PVAD Complications

Complications associated with the use of PVADs are relatively few compared with other MCS systems and primarily include bleeding, limb ischemia, cannula dislodgment, and infection [81]. The use of the Impella devices is contraindicated in patients who have heavily calcified aortic valves due to the risk of thromboembolism [82]. While the TandemHeart carries potential complications during implantation, particularly during transeptal puncture, with the risk of accidental puncture of the aortic root, coronary sinus and posterior free wall of the right atrium [81]. 

#### 3.2.3. Durable VAD Complications

Despite the clinical benefit of LVADs, with the 1-year survival rate equaling that of HTx recipients (~85%), significant complications occur with this treatment [83]. According to the INTERMACS registry, of 17,633 VAD implants, at least one adverse event occurred in up to 60% of patients by six months post-implantation, and in up to 80% by two years [58]. Major complications include bleeding, device thrombosis, infection, neurological complications (including strokes), renal impairment, and multi-organ failure [84]. Cardiac specific complications include cardiac arrhythmias, right ventricular (RV) failure and aortic insufficiency.

Ventricular Arrhythmias - Recurrent and frequent ventricular arrhythmias (VAs) are known to occur following LVAD implantation in up to 35% of patients [85,86]. They have been attributed to changes in repolarization of the ventricles due to acute LV unloading following LVAD implantation [87]. Additionally, VAs may also be caused by altered calcium handling due to the upregulation of sarcomeric and calcium handling genes, by mechanical irritation caused by the inflow cannula at the LV apex, or by lack of beta-adrenoceptor (β-AR) antagonism use [86]. Oswald et al. demonstrated that VAs occurred more commonly in patients with nonischemic HF (50%) compared to the incidence in ischemic HF patients (22%) [88]. Management of VAs in VAD patients is approached by following appropriate pharmacological treatment including β-AR antagonists and other antiarrhythmic drugs, while there is no consensus regarding the placement of automatic implantable cardioverter defibrillators (AICD) [88]. While the current pharmaceutical treatment protocol is necessary to attenuate any VAs associated with VAD implantation, this could inhibit some cardioprotective strategies while using these devices (see Section 3). 

Aortic Insufficiency - Long-term VAD support may lead to de novo aortic valve lesions, promoting commissural fusion, stenosis and aortic insufficiency (AI) [89], occurring in 10–40% of patients [90]. The mechanisms of this complication is multifactorial. Previous clinical studies have shown that the development of AI has primarily been with the HeartMate II VAD suggesting that continuous flow physiology plays a partial role in the development of this complication [90]. The comparatively small diameter of the VAD outflow cannula in relation to the aorta, results in increased fluid velocities in the aorta, causing greater wall stress [91,92]. This in turn results in thinning of the aortic medial layer, causing aortic root dilation and AI. In addition to this, LVAD patients may not be able to generate sufficient systolic pressure to open the aortic valve, thus high-pressure and velocity jets of regurgitated blood volumes contacting the root side of a closed aortic valve may result in valvular damage and degeneration [90]. Pulsatile flow can combat this complication and protect the aortic valve. Therefore, artificial pulse generated by the VAD would be necessary to facilitate this. The only device clinically available that can generate a pulsatile flow rate is the HeartMate 3, however further investigation into the operation and management of this device would be required for this application. 

Right Ventricular Failure - Right ventricular failure (RVF) is considered one of the most serious complications following left-VAD (LVAD) implantation and can occur in 9–49% of LVAD recipients in the postoperative period [93,94]. LVAD function and blood flow relies heavily on right ventricular (RV) function, thus the occurrence of RVF leads to decreased tissue perfusion and multi-organ failure [95], which are associated with increased morbidity and mortality, and reduced survival to HTx [96]. The pathophysiology of RVF post-LVAD is not well understood and the development of RVF can occur for a variety of reasons. The function of an LVAD is to provide systemic blood circulatory support to augment the failing LV and restore normal cardiac output, however, this is greatly dependent on the appropriate filling of the LV by the RV. Following implantation, initiation of LVAD support begins by slowly increasing pump speed, which decompresses the LV and increases preload to the RV [97]. This exposes the RV to loading conditions similar to those provided by a normal functioning LV, leading to an increase in RV preload; an alteration of RV contractility, and an increase in RV afterload due to pulmonary vasoconstriction, all of which can lead to RVF with any existing RV impairment [96]. This sequence of events can lead to RVF, and can start intraoperatively or in the early postoperative period [98]. 

Furthermore, the LV and RV are interdependent, with any change in compliance, shape or size of one ventricle affecting the function of the other due to the interventricular septum, interlacing muscle fibres, and the pericardium [99]. In the context of LV unloading, leftward shift of the intraventricular septum may decrease septal contribution to RV contraction, leading to RVF. In a retrospective study of 76 patients receiving a continuous-flow LVAD, persistent leftward shift of the interventricular septum 30 days post-implantation was associated with significantly worse outcomes at 90 days [100]. Determining which patients are at highest risk of developing RVF after LVAD implantation is a challenging clinical problem. Early recognition of RV failure is key to improving outcomes in these patients independent of the treatment therapy (medical therapy, MCS for the RV, or both). In order to improve clinical outcomes, altered perioperative management strategies aimed at RV protection are required, however elucidation of the mechanisms causing this complication are required before this can be realized. 

### 3.3. Cardioprotective Strategies for MCS

Cardioprotection as a field has largely centred around pharmacological treatment strategies and promoting innate protective responses aimed at preserving the function and viability of the heart following cardiac injury. However with the technological advances in MCS made in the past decade, the use of these devices to support the heart and circulatory system offer a new field of investigation into preserving cardiac function. Presented herein are potential strategies implementing MCS to preserve or improve cardiac function. Due to the nature and size of the devices, the majority of the research is physiological in nature, from either pre-clinical or clinical studies. As the field continues to expand, more mechanistic research is required to truly harness MCS as a cardioprotective strategy. 

#### 3.3.1. Acute Mechanical Unloading

Limiting myocardial infarct size and combating IRI, remain important targets of clinical treatment. This was clearly indicated by a recent study of >2600 patients showed that for every 5% increase in infarct scar size, the 1-year all-cause mortality increased by 19%, and 1-year HF hospitalization increases by 20% [101,102], when treated for primary reperfusion. Acute mechanical unloading of the heart has recently gained momentum as a clinical treatment option following acute myocardial infarction (AMI) [53]. 

Acute mechanical unloading of the heart is a reduction in the mechanical power expenditure of the ventricle, by taking blood directly from the LV to the aorta while maintaining systemic and coronary perfusion pressure, known as LV-aortic pressure uncoupling. Clinically, this can be achieved by using a PVAD, which reduces the metabolic demands and physical forces on the heart. In the context of cardioprotection, particularly in the setting of AMI, this uncoupling of the LV from the systemic circulation aims to limit cardiac power expenditure, minimize myocardial oxygen consumption (MVO_2_), protect against IRI, limit infarct size and reduce hemodynamic forces and ventricular wall stress that lead to ventricular remodeling [102,103]. 

A number of pre-clinical studies have demonstrated the efficacy of acute mechanical unloading. Meyns et al. showed that LV support using a catheter-mounted axial flow pump led to reduced MVO_2_ during ischemia and reperfusion, and reduced infarct size that correlated with the degree of unloading during reperfusion [104]. Thus, more myocardial salvage can be achieved with more ventricular unloading. In a canine MI model, the timing of LV unloading significantly impacted mitochondrial function and integrity, with normal mitochondrial integrity and ultrastructure observed if LV unloading was applied prior to reperfusion. Conversely, LV unloading commenced after reperfusion, similarly to no support, increased contraction band necrosis, mitochondrial calcium deposition and mitochondrial swelling [105]. Furthermore, the results from these studies were confirmed by Tamareille et al. who demonstrated that infarct size was reduced by 54% with LV unloading in pigs, while simultaneously reducing endothelin-1 and calcium overload, both important mediators of reperfusion injury [106]. Kapur et al. confirmed that LV unloading reduced infarct size and induced release of RISK pathway mediators, SDF-1α (cardioprotective cytokine) and antiapoptotic markers [53,107]. Further studies by this group identified increased expression of genes associated with mitochondrial integrity and cellular respiration and improved cardiac function 28 days following AMI, with limited expression of HF biomarkers [108]. 

Despite the positive pre-clinical studies in this area, the clinical application of acute mechanical unloading was limited technologically until the clinical introduction of PVADs. The Impella and TandemHeart can be implanted before or during other percutaneous coronary interventions (PCI), eliminating the need for multiple procedures to be performed on the patient. From the USpella registry, it was shown that early use of the Impella 2.5 to provide LV unloading and hemodynamic support prior to PCI was associated with more complete revascularization and higher in-hospital and 30-day survival of patients [109]. This was further confirmed in another registry study of 287 patients, also indicating improved survival to discharge over patients receiving late mechanical support [110]. However, prospective clinical studies, further coupling the clinical outcomes with the mechanisms behind acute mechanical unloading are still required. 

Acute mechanical unloading can also be coupled with V-A ECMO in order to reduce the current complication of LV distension and decrease mortality in adult patients with cardiogenic shock [111]. This is achieved by simultaneously supporting a patient with either a PVAD or IABP while under V-A ECMO support, in order to unload the heart. The use of IABP to unload LV in V-A ECMO patients (135 cases) was reported in 2014 by Gass et al. to reduce the rate of complications and mortality [112]. Blade atrial septostomy or atrial septostomy, and placement of an LA venting cannula has also been described. In addition, catheter-based LV drainage (e.g., Impella) can be used to reduce LV distension [113]. In a retrospective analysis of 66 patients, ECMO+Impella (ECPELLA) showed significantly lower 30-day mortality than the ECMO group [114]. No other secondary outcomes were observed, except high inotrope usage with ECMO alone. Likewise, another retrospective study of 157 patients reported similar findings, where patients in the V-A ECMO and Impella group had significantly lower hospital mortality and a higher success rate of bridging to recovery or further therapy [115]. In addition, there is a current RCT studying the benefit of Impella with V-A ECMO for cardiogenic shock (REVERSE, NCT03431467). This study aims to investigate if the addition of direct ventricular unloading using Impella CP leads to higher rates of cardiac recovery, defined as survival free from MCS, HTx or inotropic support at 30 days. The study is expected to complete by 2022. 

#### 3.3.2. Pulsatile Flow

The first generation of implantable LVADs mimicked the physiological action of the heart producing pulsatile flow. Current continuous flow VADs are superior to the first generation devices with their small size enabling implantation, longer durability, better energy efficiency, less surgical trauma, and reduced incidence of infection, while also having better outcomes post-HTx [56]. However, pulsatile flow offers certain advantages with less vital organ injury and systemic inflammation [116,117]. Kato et al. compared myocardial function and structure in patients implanted with pulsatile- and continuous-flow VADs [118]. They found that LV systolic and diastolic function was better in patients with pulsatile devices, having increased levels of BNP, TIMP-4 and MMP-9. Bartoli et al. reported that pulsatile flow preserved physiological values of myocardial energy utilization and vascular hemodynamics, such that when pulsatile unloading increased, normal LV pressures were maintained; however, under continuous flow, the pressure–volume relationship collapsed and the aortic valve remained closed [119]. To combat the complications associated with continuous-flow VADs, the HeartMate 3, a centrifugal pump, was designed to have intermittent speed reduction, to reduce stasis in the pump, as well as providing artificial pulsatility [120,121]. This device could be utilized as a cardioprotective strategy, targeting complications such as AI and improving LV systolic and diastolic function, as well as end-organ and microcirculatory perfusion.

Similarly to VADs, V-A ECMO patients can experience complications attributed to continuous flow. Continuous flow with V-A ECMO causes microcirculatory dysfunction [122], and is likely a key factor in contributing to coronary hypoxia. Currently, there are few ECMO systems capable of producing pulsatile flow, one example, however, is the i-cor system by Xenios. A recent study showed that pulsatile flow of this system led to improved renal function and systemic vascular tone in an adult swine model [123]. In addition, other studies have also suggested that pulsatile perfusion may help reduce systemic vascular resistance, and improve myocardial blood flow [124,125]. These benefits are likely to have a significant impact on the outcome of patients suffering cardiogenic shock, as well as other ECMO-related complications, such as acquired von Willebrand syndrome and acute kidney injury [126].

#### 3.3.3. Heparin

Studies have previously reported cardioprotective effects of heparin and its low-molecular-weight derivatives (LMWH). Their applications in AMI are well-documented [127]. Heparin and LMWH (e.g., N-acetylheparin, enoxaparin) have been shown to protect the heart against IRI in several ex vivo and in vivo animal models, with improvements in creatinine kinase, end-diastolic pressure, inflammatory responses and infarct size evident with heparin treatment prior to ischemia [128,129,130,131]. Application of heparin or LMWH also resulted in reduced activation of the complement pathway, and heparin also promotes dimerization of CXCL12, which is the cardioprotective form of CXCL12 (as compared to monomeric) [132]. The cardioprotective effect of heparin, however, is independent of its anticoagulation properties. There were clear hemodynamic complications of heparin being used as a cardioprotective agent in ECMO. In contrast, LMWH was successfully used in an observational study of >60 V-V ECMO patients [133]. Another consideration is that most of these studies were reported ~20 years ago, with very little clinical development of heparin as a cardioprotective agent over this time. Heparin plays an essential role in ECMO (and other cardiac-related interventions and surgeries) and may possesses actions that are beneficial to the heart. However, its cardioprotective effect in the context of ECMO and MCS in general is yet to be delineated. Its use must also be carefully balanced with its effect on potential bleeding complications, which is still one of the most lethal complications associated with V-A ECMO and MCS. 

#### 3.3.4. Mitochondrial Transplant

Ischemia-reperfusion injury (IRI) has long been associated with mitochondrial dysfunction. Consequently, transplantation of respiratory competent mitochondria into tissues injured by IRI has recently been proposed and tested in vitro, ex vivo and in vivo with IRI [134,135,136,137,138,139,140,141,142,143,144,145]. Mitochondria were isolated from subjects’ own skeletal muscle tissues and injected into the left ventricles. In the porcine model [138], several cardioprotective markers were observed following mitochondrial transplant, including increased extracellular myocardial ATP, intracellular ATP synthesis, up-regulation of pathways of energy generation and cellular respiration, and increased expression of cardioprotective cytokines (EGF, GRO, MCP-3 and IL-6). These molecular changes were coupled to a significant reduction in infarct size and cell death. Recently, mitochondrial transplant enabled extension of cold ischemic preservation of the donor heart [141]. Mechanistically, autologous mitochondria are endocytosed by cardiomyocytes using an actin-dependent mechanism, and improve mitochondrial respiratory function and mitochondrial DNA levels [142]. In addition, mitochondrial transplant of allogeneic and syngeneic mitochondria does not stimulate an immune response regardless of exposure frequency, or induce production of damage-associated molecular pattern molecules (DAMPs) [144]. In a recent pilot study, five pediatric patients supported by V-A ECMO for IR-associated myocardial dysfunction after surgical procedures were treated with mitochondrial transplantation [137]. These patients were exposed to myocardial ischemia after cardiac surgery that could not be improved by surgical intervention or ECMO. There were reported qualitative improvements in LV function, without observable short-term complications. However, this is still a highly controversial subject, with a very small sample size [146]. In particular, procedures performed in preclinical studies were significantly different from the clinical study where outcomes were qualitative in nature and must be progressed and interpreted with caution. 

#### 3.3.5. Mesenchymal Stem Cells

Mesenchymal stem cells (MSCs) are multipotent adult stem cells that have gained interest for their immunomodulatory potential [147]. MSCs are present in adult blood marrow and upon undifferentiation, could be induced to differentiate into a range of cell types, including adiopocytic, chondrocytic or osteocytic lineages [148]. MSCs are also known for their regenerative and repair mechanisms upon tissue and cellular damage, mainly through mitochondrial transfer in the context of brain injury, cardiac myopathies, muscle sepsis and lung injury [149,150]. However, the exact cellular signaling pathways involved in mitochondrial transfer remain to be elucidated [150,151]. Currently, one of the most promising applications for MSCs is graft versus host diseases following bone marrow transplantation [152,153]. Recently, our group has examined the effect of MSCs in ECMO, and observed an immunomodulatory effect of MSCs as expected, with reduced levels of pro-inflammatory cytokines observed [153]. However, due to the natural plastic-sticking properties of MSCs, our studies concluded it is incompatible with the current ECMO setting. It severely and irreversibly clotted the ECMO oxygenator, resulting in a rapid decline in ECMO performance [152]. Further studies aimed at improving the integration of MSCs in ECMO. In particular, a platform that allows the benefit of MSCs without direct contact of the oxygenator is warranted.

Similar to the advantages that MSCs may offer in ECMO, the combination of MSCs and long-term VAD support is an attractive concept and may offer significant advantages for myocardial recovery over using these therapeutic approaches separately. VADs have been shown to allow recovery of the heart at a molecular and cellular level; however, translation of this into clinically functional cardiac recovery, where patients are weaned from the VAD is relatively rare [154]. Thus, combining cell treatment may assist in bridging the gap between molecular and cellular recovery and restored cardiac function. Zheng et al. conducted the only pre-clinical safety and feasibility study to date [155]. This study was conducted in six sheep, combining LVAD implantation and transendocardial injections of allogenic sheep mesenchymal precursor stem cells (MPCs). Here, myocardial infarction was induced (day 0), followed by LVAD implantation (day 30) and cell injection (day 30 or 45). Five sheep tolerated all procedures and histologic analysis confirmed that the MPCs were successfully delivered; however, measurements of cardiac function were not made during this study. Clinically, Ascheim et al. Ascheim et al. evaluated the safety of intramyocardial injection of 25 million allogenic MPCs delivered at the time of LVAD implantation. They assessed the efficacy of this treatment in thirty patients with either ischemic or non-ischemic HF by evaluating LV function during short intervals of temporary reduction of LVAD support (pump speed) [156]. At 90 days post-implantation, there were no differences between the treatment and control groups for adverse events, and the treatment group showed a slightly higher rate of successful temporary weaning from the LVAD (50% vs 20%; *p* = 0.24). However, by 12 months there was no difference between groups. In the largest study to date, Yau et al. conducted a randomized trial in 159 patients, with intramyocardial injections of 150 million allogeneic MPCs [157]. The results of the trial indicated that there was no difference between the treatment and control group with respect to temporary weaning for the LVAD at 6 months and thus, the findings do not support the use of MPCs for promoting cardiac recovery at this stage.

## 4. Heart Transplantation 

Heart transplantation (HTx) is the definitive and most effective therapy for qualifying patients with end-stage HF. Donor hearts are predominantly sourced from brain dead (BD) donors; however, interest is growing in accessing hearts from patients that have died due to circulatory death (DCD donors). The demand for donor hearts continues to grow, but supply does not. Many donor hearts are discarded (>75% of donor hearts in Australia are discarded) [158], waitlists and deaths on waitlists are excessive and expanding [159], and the majority of HF patients are never assessed for HTx due to donor heart shortages and stringent recipient criteria [160]. 

Standard clinical practice using BD donors for HTx is a limitation that contributes to the high discard rate of donor hearts. Pre-existing damage, systemic factors, age, and BD-related injury in the donor can significantly prevent organ donation and increase the risk of primary graft dysfunction (PGD) in the recipient [161,162,163]. In the first 30 days post-HTx, PGD and multi-organ failure are responsible for 66% of the mortality—with most deaths attributed to PGD [163]. Our understanding of pathophysiological changes occurring with PGD are limited; however, a multi-centre survey identified IRI upon autopsy was responsible for 48% mortalities caused by PGD [163]. 

Brain death induces contractile dysfunction of the donor heart, and sensitizes the heart to IRI via the characteristic catecholamine ‘storm’, cardiac ischemia, hormonal dysregulation, mitochondrial and cytosolic calcium overload, changes to calcium sensitivity, endothelin and inflammation activation [161,162,164]. During the process HTx, the donor heart is exposed to a combination of severe injuries that are unavoidable due to the nature of the procedure: BD (and related aforementioned injuries), cold ischemic preservation whilst in transit, warm ischemia during explant and implant, and reperfusion injury following restoration of blood flow in the recipient. Although essential to viability, reperfusion of the already inflamed organ is deleterious [11,165].

**Figure 2:**
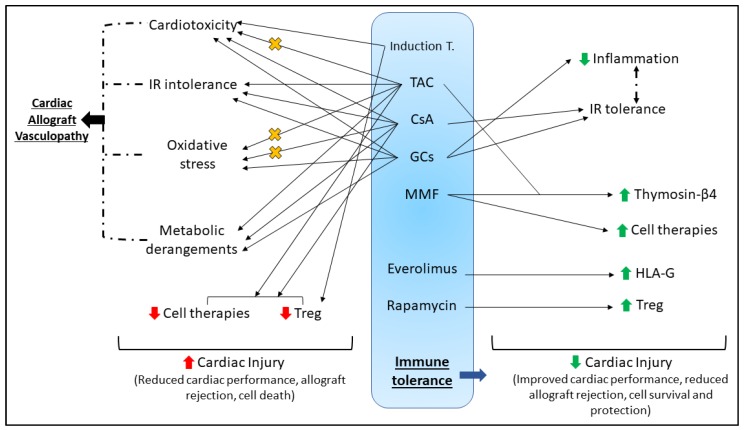
Summary of the complex effects of immunosuppression post-HTx (discussed in Section 4). The goal of immunosuppression in the recipient post-HTx is to develop immune tolerance to the cardiac allograft, thus reducing the chances of rejection and improving long-term survival. Many agents used in induction and maintenance therapy, while critical for the development of immune tolerance, have significant detrimental side effects that could complicate the efficacy of any potential cardioprotective therapeutic; including cardiotoxicity, IR intolerance, oxidative stress and systemic metabolic derangements. These adverse effects are interrelated and well reported to influence each other; notably all contribute to the development of cardiac allograft vasculopathy, worsen cardiac injury and impair patient survival. Conversely, some studies have assessed the efficacy of immunosuppressive agents upon IR tolerance, or have considered immunosuppression for the assessment of other cardioprotective strategies. Cardioprotection using cell therapy, and innate tolerance and protection induced by Treg cells appear to be abrogated by some immunosuppressive agents (e.g., CsA and TAC), yet promoted by others (e.g., MMF, rapamycin). Some agents have been shown to impede the oxidative stress and cardiac dysfunction (denoted by orange X) associated with TAC (e.g., MMF, aliskiren, almesartan) and CsA (e.g., Erdosteine). The complexities of inducing immune tolerance are worthy of important consideration for protecting the cardiac allograft and promoting survival. Induction T—induction therapy; IR—ischemia-reperfusion; TAC—tacrolimus; CsA—cyclosporine A; GCs—glucocorticoids; MMF—mycophenolate mofetil; HLA-G—human leukocyte antigen-G; Treg – T regulatory cells.

**Figure 3:**
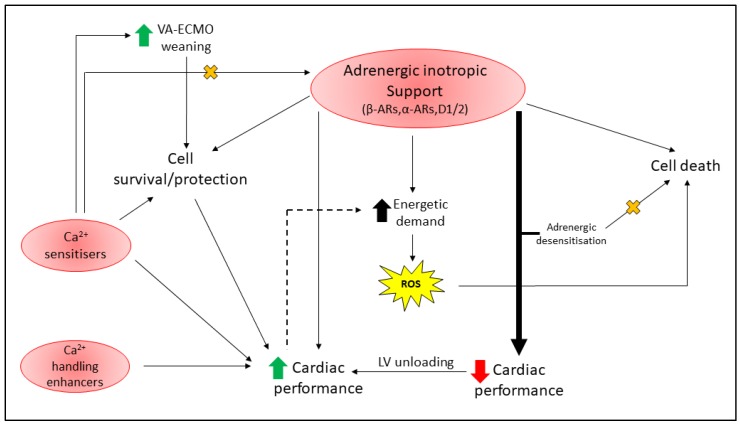
Complications of inotropic support in critically ill cardiac patients (discussed in Section 5). Adrenergic inotrope support is complex depending on the drug employed and its receptor selectivity (β-ARs, α-ARs, D1/2), with activation of the receptor pathways linked to both cell death and survival signaling. Adrenergic inotropic support is commonly used to improve cardiac performance, and while critical to patient survival, can increase the energetic demand of the already weak heart and promote reactive oxygen species (ROS) production. Elevated oxidative stress in the damaged heart can exacerbate cardiomyocyte death and worsen patient survival. Excessive adrenergic inotrope support (thick black arrow) to maintain hemodynamic control can lead to adrenergic desensitization (to prevent chronic adrenergic toxicity—denoted by orange X), at the expense of resultant poor cardiac contractility in critically ill cardiac patients (e.g., HF and post-HTx). Ca^2+^ sensitizers (e.g., Levosimendan, Omecamtiv mecarbil) appear to promote cell survival through various mechanisms, can facilitate weaning from VA-ECMO, and reduce the requirement for adrenergic inotrope support (denoted by orange X); however, they can be arrhythmogenic. Ca^2+^ handling enhancers have undergone several clinical trials and others are currently underway. Novel pharmacotherapeutics that can improve cardiac performance whilst avoiding energy imbalances and resultant oxidative stress (dotted line as is only reported to occur for adrenergic agents) are desirable in this cohort.

Following HTx, a risk of up to ~20% 90-day mortality persists, depending on the recipient’s disease state [163]. Evidently, the danger continues well beyond receiving a new a heart, and despite best practice, these injuries are currently unavoidable. Early, effective and persistent intervention is key to reduce the extent of graft dysfunction, long-term risks (e.g., cardiac allograft vasculopathy, CAV), survival and quality of life post-HTx. Cardiac dysfunction post-ischemia can be alleviated with various cardiac conditioning strategies in animal models; however, these strategies have shown mixed results clinically. Clinical cardioprotection, while desirable, has often failed due to the inherent molecular changes that occur with age, disease, and chronic pharmacotherapy [16], or due to flaws in the experimental design of both human and animal studies [14]. In the context of HTx, there are additional factors at play that should be considered to achieve effective cardioprotection in this cohort, largely immunosuppression (Figure 2) and inotrope support (Figure 3).

### 4.1. Immunosuppression

Immunosuppressive therapy is a fundamental principle in HTx recipient management against antibody-mediated and cellular rejection, but it is also a careful balancing act to avoid adverse outcomes from robust immunosuppression. Various grades of rejection and resultant graft injury can occur both short- and long-term post-HTx, thus immunosuppression protocols typically exist of induction therapy (administered at the time of transplant/early post-operative period), followed by maintenance therapy that continues for several months–years post-HTx. The International Society for Heart and Lung Transplantation (ISHLT) reports that in 2016, induction therapy was used in 76% of adult HTx recipients to reduce the risk of rejection [166]. Induction therapy commonly targets T-cell depletion (using anti-thymocyte globulins, anti-lymphocyte globulins and alemtuzumab), and inhibition of the interleukin-2 receptor (e.g., Basiliximib) [166,167]. Maintenance immunosuppression generally involves a combination of corticosteroids (e.g., methylprednisolone, prednisone, prednisolone), a calcineurin inhibitor (CNI, e.g., tacrolimus, TAC; cyclosporine A, CsA), and an antimetabolite (e.g., purine inhibitors azathioprine, AZA, and mycophenolate mofetil, MMF). Long-term, for the prevention of CAV (a common long-term complication post-HTx that impairs the coronary vasculature [168]) or the treatment of chronic rejection, a proliferation signal inhibitor (PSI) may be employed (e.g., Sirolimus, everolimus) [166,167,169].

### 4.2. Immunosuppression Can Impair Cardiac Function

#### 4.2.1. Induction Therapy

As detailed above, immunosuppression is critical for limiting rejection post-HTx, yet is not devoid of inducing deleterious effects upon patients. Induction immunosuppression using monoclonal antibody-based T-cell therapies has been associated with cytokine release syndrome (CRS). While the pathomechanisms are poorly understood, CRS develops in response to excessive T-cell activation that generates a cytokine storm leading to detrimental outcomes on all major organ systems [170]. Induction agents such as anti-thymocyte globulin (ATG) [171], alemtuzumab [172] and rituximab [173] have all been associated with CRS, and HTx recipients thus require pretreatment with antihistamines, antipyretics and glucocorticoids [167]. In response to the excessive inflammatory response with CRS, the heart may develop adverse cardiovascular complications that while reversible, appear rapidly and are severe (e.g., hypotension, tachycardia, arrhythmia, elevated troponin I release) [170,174]. 

#### 4.2.2. Maintenance Therapy 

In the past 10 years, >75% HTx recipients (at 1 year post-HTx) reported use of TAC-MMF maintenance therapy combined with a corticosteroid [166]. While key to the maintenance immunosuppression regime for HTx recipients, calcineurin inhibitors tacrolimus (TAC) and cyclosporine A (CsA) have notable cardiotoxic effects. These immunosuppressants inhibit calcineurin through the formation of specific protein complexes that effectively block T- and B-cell activation [175]. Tacrolimus is associated with hypertrophic obstructive cardiomyopathy in pediatric gastrointestinal and liver transplant patients, [176,177,178], sinus bradycardia [179], cardiac arrhythmia [180], and more recently, dilated cardiomyopathy [181]. While TAC appears to be the preferred CNI of choice over CsA [166], is it reportedly neurotoxic [167], nephrotoxic [182], induces elevated sympathetic activation [183], and does not impede the development of CAV [175,184,185,186]. CsA-mediated cardiotoxicity manifests as impaired cardiac contractility and arrhythmogenic responses, potentially due to enhanced apoptosis [187], oxidative stress [188,189] and excessive intracellular calcium sequestration [190]. Furthermore, CsA abrogates neuronal mitochondrial activity [191], leading to impaired cardiac autonomic function and hemodynamic compromise [192]. Similarly to TAC, CsA does not lower the incidence of CAV. This may in part be linked to the observation that clinical doses of CNIs may not completely abrogate calcineurin, leading to partial immune system activation and resultant development of CAV [193]. Both TAC and CsA are associated with the development of metabolic derangements. In comparison to TAC, CsA appears to potentiate hyperlipidemia and hypertension post-HTx more, two risk factors that contribute to CAV development. Tacrolimus has also been associated with the development of new-onset diabetes and impaired glucose tolerance following both heart and renal transplant [175,184,194,195,196,197,198]. Despite these cardiovascular and metabolic complications, TAC and CsA have notably improved medium-term longevity and are equally effective at limiting acute rejection post-HTx [184,194,199]. Immunosuppressive therapy is quintessential to management and recovery post-HTx, however it is clear that both metabolic and cardiac dysfunction may not be avoided in some patients.

Glucocorticoids (GC, e.g., prednisone, methylprednisolone, prednisolone) are a core component of maintenance immunosuppressive treatments to promote immune tolerance post-HTx. Mechanistically, GCs bind cytoplasmic glucocorticoid receptors, and beneficially regulate inflammation and immunosuppression by both transcriptional and non-transcriptional pathways [169]. To reduce inflammation, GCs have been shown to attenuate leukocyte adhesion to the endothelium, and adhesion molecule expression of endothelial cells [200]. Furthermore, GCs reduce cytokine expression at a transcriptional level, and block macrophage/monocyte infiltration [201]. While it appears that mechanistically, GCs may offer a protective advantage in the heart, several studies report that GCs worsen outcomes from IRI [202,203,204,205,206]. Indeed, a review of clinical trials and meta-analyses in 2014 revealed that despite the distinct anti-inflammatory actions of GCs, no clear benefit to mortality or incidence of myocardial infarction was identified by any clinical trial [200]. Regardless, steroids remain critical to immune tolerance, and are thus an important component of immunosuppressive induction, maintenance and rejection therapies [169,207]. Interestingly, faster withdrawal of steroid therapy does not impact early rejection post-HTx [208,209]. Steroid withdrawal is a desired goal, as long-term steroid therapy is well-reported to be associated with significant comorbidities such as diabetes, obesity, hyperlipidemia and hyperglycemia [169,210,211,212]. These risk factors undoubtedly aggravate the incidence of CAV, which affects almost 30% patients within 5 years post-HTx, and increases to 47% within 10 years [166]. Steroid use also reportedly impairs wound healing [169,203], is associated with left ventricular free wall rupture in AMI [203], and is prothrombotic [213]. While outside the direct scope of this review, metabolic risk factors and disorders unequivocally ameliorate many cardioprotective mechanisms in both animal models and clinical studies, reviewed in detail elsewhere [13,16,214,215]. 

### 4.3. Cardioprotection and HTx

During HTx, the grafted heart is unavoidably exposed to extended IRI, which can significantly impede post-HTx cardiac function in the recipient, and enhance post-HTx morbidity and mortality. Many research groups have examined variations of cardioplegic solutions to precondition the donor heart upon retrieval, in an attempt to limit IRI with varied success [216,217]. Novel avenues to prolong the time a donor heart can be stored without aggravating post-HTx graft function are being actively investigated and used clinically with notable success [218,219,220,221,222]. While these are important components within the process of HTx, they are outside the scope of this review. 

#### 4.3.1. Intrinsic Immune Responses Aid Cardiac Recovery

Despite significant immune and cardiovascular challenge, intrinsic responses remain active in the heart post-HTx. Early post-HTx, strategies aiming to shift the balance away from graft destruction and effector T cells, but towards graft regulation and regulatory T (Treg) cells is clinically desirable [223]. Regulatory immune cells contribute to immune tolerance, leading to reduced graft rejection and improved long-term survival, and Treg cell promotion can be manifested through specific immunosuppression [224], cellular therapy [223] or alloantigen pretreatment [225]. In a mouse model of myocardial IRI, Xia et al. showed that Treg cells aid in myocardial functional recovery following IRI by activation of RISK-associated Akt and ERK1/2 signaling to reduce cardiomyocyte apoptosis, and suppression of chemokine production to limit neutrophil infiltration via a CD39-dependent mechanism [226]. In a model of heterotopic heart–kidney cotransplantation where recipients received CsA, cardiac allograft function, survival and ultrastructure were not impacted despite significant inflammatory, IR and histoincompatability challenges [227]. The preserved cardiac function and allograft survival was attributed to the development of immune tolerance, which occurred despite the excessive proinflammatory settings. 

#### 4.3.2. Cardioprotection Despite Immunosuppression

While compulsory immunosuppression can impair myocardial function, some pharmacotherapies (including some immunosuppressants) retain their ability to induce a protected cardiac phenotype. Myocardial and/or soluble human leukocyte antigen-G (HLA-G) expression has been shown in several studies to reduce cardiac allograft rejection and vasculopathy post-HTx [228,229]. Expression of HLA-G appears more prominent in HTx recipients receiving everolimus (a PSI) compared to mycophenolate mofetil (MMF), and this expression is not affected by CsA treatment [230]. Alternatively, the antioxidant properties of MMF can reduce the oxidative stress and DNA damage induced by TAC [231]. Treatment with an angiotensin receptor blocker (almesartan) or a renin inhibitor (aliskiren) limited cardiac injury biomarker (lactate dehydrogenase, creatine kinase) expression in serum, preserved cardiac structure and effectively reduced oxidative stress in TAC-treated hearts [232]. Targeting oxidative stress, erdosteine (a mucolytic with antioxidant properties) co-treatment with CsA for 21 days reduced the myocardial structural derangements, interstitial fibrosis and improved the oxidant/antioxidant balance [189]. While the aforementioned studies show that immunosuppression complications may be rescued with other pharmacotherapies, Behbod et al. demonstrate that inhibition of Jak3 using tyrphostin AG-490 potentiates the beneficial immunosuppressive actions of CsA and extends cardiac allograft survival [233]. Additionally, CsA is capable of reducing troponin I and creatinine kinase release following reperfusion [234]. However, clinical outcomes in myocardial infarction patients were no different to placebo, and cardiac remodeling persisted when CsA was delivered prior to [235] or at reperfusion [236]. Ikeda et al. demonstrated that when CsA was linked to nanoparticles and administered intravenously at reperfusion, CsA improved myocardial functional recovery, reduced cardiac remodeling, and was more effective at inhibiting mPTP opening [237]. 

Despite several reports that GCs worsen IRI outcomes (detailed above), GCs also protect the heart against IRI by restoring post-ischemic contractile function [238], reducing infarct size [239,240], cardiac troponin T [241] and I [242], can prolong cardiac allograft survival [241,243], and these positive effects may be improved with simvastatin co-treatment [241]. Mechanistically, GC-mediated tolerance to IRI (and associated inflammation) reportedly inhibits of NFκB [244,245], activates endothelial nitric oxide synthase (via a PI3K/Akt-dependent pathway) and annexin-1 [169,245], and modulates angiotensin II receptor subtype expression [238]. Furthermore, exposing hearts to dexamethasone 24 hours prior to IR increases expression of cyclooxygenase 2 (COX-2) [246] and activates lipocalin-type prostaglandin D (PGD) synthase [246,247], with converging end effects to increase prostaglandin D_2_ production (PGD_2_) [248]. Elevated PGD2 expression limits oxidative stress by increasing Nrf2 transcription factor levels [248,249]. 

Methylprednisolone (MP) is the primary GC of choice for use in HTx recipients [166]. Donor BD induces significant RV dysfunction and pro-inflammatory cytokine upregulation, which is reportedly attenuated with MP treatment either early pre-or post-BD induction [250,251]. Altering the balance of the pro- and anti-inflammatory profile with MP administration is not influenced by NFκB or β-adrenoceptor signaling pathways [251]. Furthermore, reduced IL-8 and TNFα, and increased anti-inflammatory IL-10 were observed when steroids were administered earlier in the immunosuppressive protocol to protect against CPB-induced inflammation [252]. Sandha et al. have recently demonstrated that MP is also beneficial in DCD settings, effectively reducing pro-inflammatory cytokines in DCD hearts with MP delivery at reperfusion [253].

Akin to many cardioprotective agents, the timing of steroid delivery likely influences the degree of protection. Wan et al. demonstrated that MP administration 1 h prior to cardiac surgery is more effective at reducing the pro-inflammatory profile compared to delivery at the end of CPB [252], and administering MP in the CPB prime provides no additional cardioprotection (identified by expression of heart-type fatty-acid-binding protein) vs. induction of anesthesia [254]. A human study found that significantly reducing the steroid dose during BD donor management did not worsen donor heart function or rate of successful HTx, and preserved glucose handling [255]. Steroids administered immediately prior to HTx improved post-HTx cardiac contractility and hemodynamic stability [256].

#### 4.3.3. Cardioprotective Cellular Therapies and Immunosuppression

Cardiovascular cellular therapies and regenerative medicine have shown promise in alleviating infarction and restoring post-ischemic function in many animal models. In the context of HTx and associated immunosuppression, several cell therapies identified have improved cardiac allograft function and survival. Indeed, in some circumstances, correct immunosuppression choice has improved cell engraftment with resultant beneficial effects on the heart. Huber et al. [257] have previously shown that an alternative short-course immunosuppressive regime using two co-stimulation adhesion inhibitors (CTLA-4 and anti-LFA-1) permitted enhanced survival of human embryonic stem cell-derived endothelial cells (hESC-EC) and cardiomyocytes (hESC-CM) in the ischemic mouse heart. Furthermore, improved hESC-EC engraftment following this immunosuppressive treatment significantly improved post-ischemic cardiac function and reduced infarct size [257]. Conversely, proliferation and quantity of hESC-CM is reduced by clinically-relevant doses of CsA and TAC [258]. In a heterotopic HTx model where recipients were treated post-operatively with TAC and MMF, gene transfer of thymosin β4 (a G-actin-sequestering molecule associated with cell survival, immunomodulation, improved myocardial function and infarct reduction [259,260]) improved post-HTx survival and reduced graft rejection through anti-inflammatory, pro-angiogenic and improved cardiomyocyte survival mechanisms [261]. Mesenchymal stem cells (MSCs) have gained significant attention for their potential as a therapeutic for cardiac disease, demonstrating functional tolerance and protection against infarction following cardiac ischemia [262,263,264,265]. They also possess immunomodulatory actions as activators of Treg cells, demonstrating their potential benefit in settings of clinical HTx. Cell therapy using MSCs reportedly induces long-term allograft acceptance when applied with MMF [266]. However, this beneficial effect is not only lost in the presence of CsA, but low-dose CsA also promoted allograft rejection in these settings [267]. 

Due to the role of Treg cells in promoting graft tolerance post-HTx, development of a Treg-based therapy that could reduce the reliance upon chronic immunosuppression while promoting allograft tolerance is clinically desirable [268,269,270,271,272]. Antigen-specific Treg cells (isolated from T-cell receptor transgenic mice) appear superior to polyclonal Treg cells due to their enhanced nonspecific suppressive function at sites of localized inflammation [273,274,275], such as the cardiac allograft post-HTx. Pilat et al. previously demonstrated that additional Treg treatment at the time of bone marrow transplantation in the recipient (performed 6–8 weeks prior to heterotopic HTx to precondition the recipient with fully mismatched donor bone marrow cells) prevents the development of chronic rejection in cardiac allografts [276]. While Treg-based therapies may be useful in promoting cardiac allograft survival, Treg activity is modified by immunosuppression. Rapamycin promotes functional Treg cell numbers in vivo and in vitro [277,278] and assists in promoting long-term cardiac allograft survival following alloantigen-specific Treg cell treatment [275]. However, CNIs [279], alemtuzumab and ATG [280] reportedly reduce Treg populations. 

Immunosuppression is an important component of recipient management to enhance post-HTx recovery and survival. However, these drugs have well-reported adverse effects upon the heart (and rest of the body) that complicate post-HTx recovery. Indeed, a recent study assessing the ISHLT registry data found that recipient age and immunosuppressive therapy likely influence post-HTx mortality, proposing that immunosuppression should be tailored to the recipient age [281]. While some cardioprotective therapies have shown promise at positively influencing post-HTx myocardial function and survival, co-treatment immunosuppression can limit their protective efficacy. Consideration of the target cohort (e.g., age, comorbidities, drug therapies) is vital to find an effective cardioprotective strategy, and in the context of HTx, immunosuppressants, should also be considered.

## 5. Cardioprotection With Inotropic Support 

The therapeutic goal of inotropic agents is to increase cardiovascular performance whilst improving end organ perfusion. Inotrope support is required for hemodynamic management of critically ill patients—including BD donors, cardiac patients following surgery (e.g., HF management, post-VAD implantation and post-HTx), and ECMO patients [282,283]. For a comprehensive review, Maack et al. discussed the use of inotropic agents in the context of HF in a position statement by the European Society of Cardiology [284]. Interestingly, many of the pathways and signaling components activated by inotropes have been linked to cardioprotective mechanisms across model systems and species. Despite this positive link, long-term inotrope support is associated with poor survival and cardiovascular outcomes [282,284,285]. Currently, these agents can be loosely classified into three main arms based on their mode of action (discussed below); adrenergic stimulants, myofilament calcium sensitizers and agents that improve cytosolic handling independent of adrenergic signaling [284].

### 5.1. Adrenergic Stimulants

Adrenergic stimulants, including adrenaline, noradrenaline, dopamine and dobutamine, are the most frequently used inotropic agents available to the clinician (Table 1). Adrenergic stimulants activate beta-adrenergic (β_1_/β_2_-ARs), alpha-adrenergic (α_1_-ARs) and dopaminergic receptors (D1-/D2-like). Commonly used in combination targeting myocardial β_1_-ARs and vascular α_1_-ARs, the use of these inotropes constitutes a careful balancing act between therapeutic action and cardiovascular compromise.

#### 5.1.1. Beta-Adrenoceptors

β_1_-adrenoceptors (β_1_-AR) are the most prominent adrenoceptor in the heart, and activation of this pathway to improve cardiac performance and survival may come at a cost. Mechanistically, inotropes used to stimulate this pathway (e.g., dobutamine, adrenaline, noradrenaline) can increase intracellular calcium overload, cardiomyocyte apoptosis, myocardial oxygen demand, lactate production and incidence of arrhythmias [282,284,286]. These adverse consequences become particularly important for patient management following myocardial infarction, where in the case of cardiogenic shock, inotropic support via adrenergic stimulants can potentially worsen prognosis [284]. Long-term use in HF patients is also potentially detrimental and perhaps ineffective due to the impaired β-AR signaling that occurs in HF [287]. Furthermore, chronic isoproterenol administration has been repeatedly used in animal models to induce myocardial damage [288,289,290,291]. While β-AR stimulants are vital to elevate cardiac performance, equally, their use can impair cardiac function. 

Stress cardiomyopathy, or takotsubo (TS) syndrome, shares similar features with the cardiac dysfunction commonly seen during donor BD: excessive catecholamine overload on the heart, and (somewhat) reversible contractile dysfunction [292]. TS develops in response to elevated circulating and postganglionic catecholamines [293], and further exogenous catecholamine administration acts synergistically to impair cardiac function and can increase mortality [294]. Models of TS using isoproterenol have highlighted the importance of β_2_-ARs in the generation of TS [295,296], which involves a switch in Gs- to Gi-coupling in the stimulus trafficking process [293,296]. Coupling to Gi increases p38 MAPK and PI3K/Akt signaling, inducing a fall in contractility, attempting to protect the heart against catecholamine toxicity [296,297]. This β_2_-AR-Gi cardio-inhibitory effect can be exacerbated by β-blockers known to have β_2_-AR-Gi agonsim such as propranolol, and to a lesser extent, carvedilol [296]. Neither drug, however, increased overall mortality in TS. The classical TS morphology is due to the regional differences in β_2_-AR expression, decreasing in density from the apex (highest) to the base (lowest) [296,297]. The denervated heart post-HTx is reportedly hypersensitive to β-AR stimulation [298,299], even in the presence of β-blockade [299]. This hypersensitivity reportedly occurs in response to reduced β-AR density over time post-HTx [298,299]. Gilbert et al. also showed that this hypersensitivity was presynaptic, and myocardial interstitial epinephrine levels were likely elevated due to an absent neuronal uptake mechanism [298]. These collective observations are in agreement with IR studies demonstrating reduced β-AR density and elevated GRK2 levels post-ischemia [300,301,302]. 

The role of β-agonism in cardioprotection is controversial, with many studies reporting protective and deleterious actions of both β_1_- [303,304,305,306,307] and β_2_-adrenoceptors [306,307,308,309,310] (β_1_- and β_2_-Ars, respectively) in the context of myocardial IRI. β-agonism that promotes Gs-PKA pathway activation is typically associated with myocardial damage, and activation of the Gi-ERK pathway is prosurvival; however, there are exceptions to this rule [305,308]. These mixed effects may reflect cardiac vs. extra-cardiac responses of β-ARs, variances in drug selectivity for β-AR subtype, and non-specific actions of β-AR responses [303]. Interestingly, a unique cardioprotective phenomenon termed sustained ligand-activated preconditioning (SLP) induces potent tolerance to IRI through regulation of the β_2_-AR-Gα_s_-PKA signaling pathway [308]. Distinct from traditional cardioprotective stimuli, these unique signaling mechanisms render SLP protective in the aged [311] and comorbid [312] myocardium, which is also insensitive to chronic β-blockade [303], caveolin-3 knockout [27]. 

#### 5.1.2. Alpha-Adrenoceptors

Cardiac α-adrenoceptor (α-AR) activation by adrenaline (or epinephrine) or noradrenaline (or norepinephrine) leads to downstream release of intracellular calcium and protein kinase C activation, resulting in vasoconstriction [313]. Increasing evidence now suggests that α1-AR activation can increase myocardial contractility, and chronic α1-AR stimulation initiates adaptive responses to protect from myocardial stress. Sustained α1-AR activation reportedly induces physiological hypertrophy that does not impair cardiac function, nor produce fibrosis [313,314,315]. In various cell and animal models, α1-AR activation can ameliorate apoptosis and necrosis induced by IRI [316,317,318], hypoxia [319], norepinephrine- [320] and isoproterenol-induced cardiotoxicity [319]. Mechanistically, this survival signaling likely involves ERK1/2 and Bcl-2 (and related family members), to preserve mitochondrial membrane integrity and increase expression of transcription factors that promote cardiomyocyte survival (GATA4 and NFAT) [313]. In the heart, α1-ARs comprise approximately 10% of total cardiac adrenergic receptor population [313]. Thus, while α1-AR signaling may be cardioprotective, adrenergic stimulants that may activate α1-AR also bind β-ARs that form the largest myocardial adrenoceptor population, and adverse β-AR-mediated consequences likely dominate when treating critically ill patients. 

#### 5.1.3. Dopamine Receptors

Dopamine receptors can be classified into two superfamilies, D1- and D2-like receptors that have either direct (D1-like) or indirect (D2-like) vasodilatory effects on smooth muscle. Both receptor subfamilies are found in vascular and cardiac tissue, with D1-like receptor activation increasing adenylyl cyclase (AC) activity and downstream calcium channel activation, and D2-like receptors inhibiting this pathway [321,322,323]. The ligand dopamine, however, has complex dose-dependent actions. At low doses (≤5 μg/kg/min), dopamine induces vasodilation via D1- and D2-like receptors. Low-dose dopamine support in organ donors limits the risk of right heart failure (pediatric recipients) [324] and three-year mortality (adult recipients) post-HTx [325]. At high concentrations, dopamine activates β- and α_1_-ARs, causing positive chronotropy, inotropy and vasoconstriction [282]. While dopamine demonstrates important roles in maintaining cardiac output and systemic perfusion, its actions are not without untoward effects. Dopamine exaggerates intracellular calcium levels following IR, such that when coupled with impaired post-ischemic calcium cycling, this leads to compromised ventricular relaxation and cardiomyocyte apoptosis, despite enhanced systolic function [326]. Interestingly, propofol postconditioning reportedly allows preservation of dopamine-induced inotropy, but ablates the associated cardiomyocyte apoptosis [326]. In clinical situations where neuronal norepinephrine stores are depleted (e.g., post-HTx following surgical denervation or end-stage HF), the effectiveness of dopamine to provide inotropic support may be compromised [327]. 

Dopamine has also been implicated in mechanisms of cardioprotection. Pharmacological preconditioning with dopamine improves post-ischemic functional recovery and reduces infarct size in a dose-dependent manner via α_1_-ARs [328] and dopamine D2 receptors [329]. Indeed, activation of dopamine D2 receptors is reportedly involved in mechanisms of ischemic pre- [329] and postconditioning [330]. The D2 receptor-mediated cardioprotection that occurs with ischemic postconditioning has been shown to mediate translocation of protein kinase C-ε (PKC-ε) to the cell membrane [331], and inhibit mPTP opening via activation of ERK1/2, PI3K-Akt-GSK3β, and PKC-ε [332]. Interestingly, N-octanoyl dopamine (NOD), a dopamine derivative, has been shown to protect against cold ischemic injury in neonatal rat cardiomyocytes [333] and in transplanted rat hearts exposed to NOD during donor brain death [334]. While NOD preserves cardiac contractility [334,335], mechanistically, NOD appears cardioprotective via its lipophilic and antioxidant properties [333,334], and has the ability to reduce the expression of apoptotic and pro-inflammatory markers in cardiac tissue [335]. 

### 5.2. Complications with Adrenergic Inotrope Support

#### 5.2.1. Adrenergic Desensitization

Following repeated stimulus exposure (e.g., as occurs in HF, BD, high inotrope support), adrenergic desensitization occurs to alter both receptor function and expression. Here, G-protein coupled receptor kinases (GRKs), particularly GRK-2, mobilize to the cell membrane and phosphorylate agonist-occupied ARs, preventing further receptor activation. β-arrestins are then recruited to the site, promoting G protein uncoupling and eventual receptor internalization [336]. Adrenergic desensitization prevents chronic adrenergic toxicity, albeit at the expense of contractility in settings of HF [337] and following HTx [292]. 

In the context of organ donation following BD, the desensitization process is of particular importance. A defining feature of BD is the catecholamine storm, increasing post synaptic release of adrenaline and noradenaline in an attempt to counteract impaired cerebral perfusion, leading to a severe hyperdynamic cardiovascular response in donors [338,339]. It is hypothesized that this catecholamine overload potentiates adrenergic desensitization in donor hearts, and promotes myocardial dysfunction. Inotropic support is vital for management of the hemodynamic compromise during donor BD, however, in a potentially desensitized organ, additional exogenous catecholamine exposure may only add insult to injury. Indeed, high inotrope dependence and significant myocardial dysfunction can prohibit organ donation (based on medical grounds) [340,341]. Due to the shortage of acceptable donor hearts, several studies have demonstrated that using these “extended criteria” donors on high inotrope support is an acceptable strategy for expanding the donor pool [342,343,344]. Conversely, left ventricular mechanical unloading (as occurs with VAD implantation, V-A-ECMO) increases β_1_-AR [345] and β_2_-AR mRNA expression (also tolerance to IRI) [346], and reduces GRK2 expression and activity [347,348], which may explain the improved β-AR responsiveness and total β-AR density observed post-VAD implantation [347,348,349]. Several studies have demonstrated restoration of improved cardiac β-AR signaling following LVAD support, Post-HTx patient management also requires inotropic support, commonly employing adrenergic stimulants. Similar to HF, however, HTx recipients may face the deleterious effects of desensitization. Studies have shown that exposure to permanent LAD occlusion or IRI (as occurs with organ storage and subsequent HTx) induce reductions in adrenergic receptor density [300,301,350]. Indeed, adrenergic stimulation at reperfusion further aggravates reperfusion injury by causing calcium overload, leading to apoptosis [351]. Increasing evidence demonstrates that GRK2 is an important signaling molecule independent of the desensitization process that can impair cardiac function, and strategies disrupting GRK2-mediated signaling appear cardioprotective [352,353,354]. GRK2 is upregulated in sepsis, HF, BD and post-ischemia [355,356,357], and translocation of GRK2 to the mitochondria can influence fatty acid oxidation and ATP production rate [354,358]. The precise effects, however, are still under scrutiny due to the variability in observations, which may be influenced by the physiological setting and cell type. 

#### 5.2.2. Energetic Imbalance

An often overlooked and misunderstood factor of adrenergic inotropic support is their energetic cost. In settings where this energetic imbalance is already disrupted in the heart due to IRI (e.g., cardiac surgery, HTx), inotrope support may exacerbate this imbalance, with inoconstrictor agents potentially more detrimental than inodilators. Increased LV afterload, coupled with elevated heart rate following inconstrictor administration significantly increases myocardial oxygen consumption rate, potentially hastening cell death [285]. Under normal circumstances, mitochondrial respiration is stimulated by calcium activation of the Krebs cycle, upregulating NADH production. Intracellular calcium overload, as a consequence of high or prolonged inotrope use, promotes mitochondrial dyscoupling and ATP depletion. Using a sheep model of 24 h BD followed by orthotopic HTx, our group has observed elevated complex I and reduced complex II-mediated mitochondrial respiration post-HTx. The increase in complex I respiration, however, occurred in the presence of higher electron slip, and did not result in production of a mitochondrial membrane potential greater than controls [unpublished data] [359]. This greater oxygen utilization in the absence of membrane potential generation implies increased ROS and lower ATP production rates. In HF, mitochondrial calcium uptake is impaired, which promotes ROS production, and subsequently potentiates arrhythmias and cardiomyocyte damage [360]. Myocardial metabolic inflexibility is also a common feature in critical illness and in response to high catecholamine exposure [361]. Due to the enhancement of carbohydrate metabolism, β-oxidation usually declines with an increase in cellular uptake of fatty acids, resulting in uncoupled mitochondrial respiration [362]. Any increase in carbohydrate metabolism will also increase the rate of lactate production, and in the context of poor calcium handling and oxidative stress, may lead to cellular acidosis and apoptosis. Theoretically, increases in glycolytic rate increase the oxygen efficiency of the cell. However, it has been shown in human and animal HF models that carbohydrate oxidation and its contribution to ATP are decreased [361]. 

### 5.3. Myofilament Calcium Sensitisers

Myofilament calcium sensitizers (e.g., levosimendan, pimobendan, EMD-57033, GCP-48506, omecamtiv mecarbil) increase the affinity of troponin C or actin-myosin to bind calcium, or directly bind to the motor domain of myosin, producing elevations in inotropy and systolic ejection time [284]. Calcium sensitizers were thought to offer several cardioprotective advantages over adrenergic stimulants. Independent of adrenergic stimulants, this group of drugs do not alter calcium handling, chronotropy or blood pressure, and thus do not induce desensitization nor impose energetic compromise. Conversely, myofilament calcium sensitizers are arrhythmogenic and reduce diastole [284]. 

Evidence thus far on the benefits of using calcium sensitizers in place of adrenergic stimulants has not yet reached a definitive conclusion. Levosimendan reportedly improves cardiac contractility and induces vasodilation without increasing the myocardial oxygen demand [363]. Furthermore, levosimendan appears to influence mitochondrial potassium ATP channels, promotes prosurvival ERK1/2 signaling [364], is anti-inflammatory and anti-apoptotic [365]. Clinical trials and metanalyses have suggested the use of levosimendan for HF reduced the relative risk of mortality, improved hemodynamics, and reduced post-operative inotrope use [284]. Beiras-Fernandez and colleagues showed that post-HTx, a 24 h infusion (0.1 μg/kg/min) of levosimendan reduced the inotropic demand and improved ventricular performance [366]. Levosimendan has also been shown to reduce the requirement for high inotrope support and facilitate weaning from V-A ECMO [367], and ameliorate pre-VAD implantation hemodynamic compromise and assist in predicting post-VAD RV failure [368]. However, other clinical trials (LeoPARDS [369], CHEETAH [370], SURVIVE [371] and LEVO-CTS [372]) have found no reduction in patient mortality, with an increased risk of tachyarrhythmias. Studies examining omecamtiv mecarbil (OM) in HF, revealed that although effective at increasing inotropy, OM also reduced diastolic time [284]. This led to myocardial ischemia, elevated plasma troponin and ECG changes, potentially a result of poor perfusion, as diastolic time was reduced in favor of increased systole. Further studies targeting specific plasma concentrations or restraining the maximum OM dose have shown improved exercise tolerance and dyspnea relief [284]. Moreover, levosimendan administered in patients who also received prior β-blocker therapy showed improved short-term outcomes in the SURVIVE trial [371]. These results, similar to adrenergic stimulants, may highlight the complexity of calcium sensitizers to their dose and clinical scenario. 

### 5.4. Enhancers of Cytosolic Ca^2+^ Handling

These drugs have multiple mechanisms of action to improve cytosolic calcium handling and are an active area of research, with ongoing trials currently underway to ascertain their clinical utility [284]. The initial CUPID 1 trial that attempted to restore myocardial SERCA2a expression and activity in HF patients via gene therapy demonstrated safety and potential efficacy [373], leading to the CUPID 2 trial. Unfortunately, the primary outcome of CUPID 2 (time to re-hospitalization) was not improved, with questions rising over the efficacy of the gene delivery [374]. The neutral results of the CUPID2 trial led to suspension of the SERCA-LVAD (NCT00534703), which was assessing AAV1.SERCA2a in HF patients who had received an LVAD, and was targeting weaning of patients from the LVAD.

Nitroxyl reportedly improves myocardial calcium transients and cardiac contraction independent of cAMP/cGMP-mediated pathways [375], and nitroxyl donors have been (CXL-1020) [376] and are currently being (CXL-1427, NCT02157506, NCT02819271) [284] investigated in Phase I and II clinical trials. Here, CXL-1020 improved myocardial function in patients with systolic HF, adult mouse cardiomyocytes, and in in vivo canine models of HF [376]. Istaroxime inhibits the Na^+^/K^+^ATPase and activates SERCA, with resultant beneficial effects on inotropy and lusitropy. In the HORIZON-HF trial, systolic blood pressure, diastolic function and cardiac index were improved with istaroxime treatment (6 h i.v.) [377], which has led to an ongoing trial in patients with acute decompensated heart failure (NCT02617446). Evidently, several drugs acting to improve cytosolic Ca^2+^ handling while avoiding cardiovascular compromise and energetic imbalances show promise in HF settings over traditional inotropic agents. Larger clinical trials are necessary to solidify their cardioprotective effects and clinical utility in critically ill cardiac patients that could benefit from an effective therapy. This is a common feature of most inotropes and vasopressors, where clinical need has circumvented empirical-based investigation into appropriate administration. Significant future research is necessary in order to determine the correct inotrope, vasopressor or combination for a particular disease state at a particular dose. This will help to delineate the balance between cardioprotection and myocardial compromise often observed during hemodynamic management.

## 6. Conclusions

Cardiovascular disease and HF continue to expand and are currently the number one cause of global mortality, despite significant advances in technology and therapeutic strategies targeting the associated cardiac dysfunction. While some treatment options are beneficial and essential for patient survival and restoration of cardiac function, their use comes at a physiological cost that can worsen both short- and long-term outcomes. Evidently, this cohort of critically ill cardiac patients would significantly benefit from complimentary and effective cardioprotective strategies. However, in the context of these relevant surgical interventions (MCS and HTx), important factors inherent to these treatments must be carefully considered to achieve clinical efficacy. 

## Figures and Tables

**Table 1:** Adrenergic stimulants used, and their receptor target affinities and therapeutic aim.

Drug	Receptor Target				Therapeutic Aim
	α_1_-AR	β_1_-AR	β_2_-AR	D1/2	
**Adrenaline**	++++	+++	++++	0	Vasoconstriction Positive inotropy and chronotropy
**Noradrenaline**	++++	+++	+	0	Positive inotropy and chronotropy Vasoconstriction
**Dobutamine**	++	+++	+	0	Positive Inotropy and chronotropy
**Dopamine**	+++	++	+++	++++	Vasodilation Positive Inotropy and chronotropy

## Abbreviations

HF	Heart Failure
HTx	Heart Transplant
BD	Brain dead
PGD	Primary graft dysfunction
IRI	Ischemia-reperfusion injury
IR	Ischemia-reperfusion
MCS	Mechanical circulatory support
VAD	Ventricular assist device
ECMO	Extracorporeal membrane oxygenation
V-A	Veno-arterial
PVAD	Percutaneous ventricular assist device
IABP	Intra-aortic balloon pump
LV	Left ventricle
RV	Right ventricle
RVF	Right ventricular failure
AMI	Acute myocardial infarction
PCI	Percutaneous coronary intervention
ICT	Intracardiac thrombosis
VA	Ventricular arrhythmia
AI	Aortic insufficiency
MSC	Mesenchymal stromal cells
MPC	Mesenchymal precursor cells
TAC	Tacrolimus (TAC)
CsA	Cyclosporine A
MMF	Mycophenolate mofetil
AZA	Azathioprine
Treg	Regulatory T cells
CRS	Cytokine release syndrome
GPCR	G protein-coupled receptors
ATG	Anti-thymocyte globulin
LMWH	Low molecular weight derivatives
HLA-G	Human leukocyte antigen-G
PGD	Prostaglandin D
PGD2	Prostaglandin D2
MP	Methylprednisolone
β-AR	β-adrenoceptors
α-AR	α-adrenoceptor
GC	Glucocorticoids
